# Dr. Kennedy on the Management of Blisters

**Published:** 1829-04-01

**Authors:** 


### LIII.

### DR. KENNEDY ON THE MANAGEMENT OF BLISTERS.

"Previously to blisters being applied, the parts should be well washed with warm water and soap, and afterwards submitted to brisk friction with a cloth that is dry, rough and hot, or well moistened with heated vinegar or spirits. If reddening and excitement only of the surface be desired, the blister ought to be removed in about five hours:—in eighteen or twenty-four it will have produced its complete effects. When, from a particular state of the system, an extreme activity of the blistering ingredients, or from their being allowed to act for an undue length of time, a deep ulcer becomes the result, this is usually slow of healing, and emits a copious purulent discharge, which tends greatly to debilitate a sick person, and to protract an otherwise favourable convalescence. Experienced parents will easily recollect having observed, and been made unhappy by, such an occurrence after blistering children:—it may be prevented, however, by determining the degree of this operation, not by mere *time*, but by the progress of its *effects*. In young children, all blisters ought to be removed immediately on the skin being uniformly reddened; after which, a warm poultice will secure for the little one all the advantages, and, at the same time, save it from many of the distresses a more palpable vesication would have induced."

